# Evaluation of anti-stigma social marketing campaigns in Ghana and Kenya: Time to Change Global

**DOI:** 10.1186/s12889-021-10966-8

**Published:** 2021-05-08

**Authors:** Laura C. Potts, Claire Henderson

**Affiliations:** 1 Department of Biostatistics & Health Informatics, King’s College London, Institute of Psychiatry, Psychology and Neuroscience, London, SE5 8AF UK; 2 Health Service and Population Research Department, King’s College London, Institute of Psychiatry, Psychology and Neuroscience, London, SE5 8AF UK

**Keywords:** Social marketing, Stigma, Low and middle income countries

## Abstract

**Background:**

Launched in 2018, Time to Change Global is a new anti-stigma programme to tackle stigma and discrimination towards people with mental health problems in low- and middle-income countries. Our aim was to evaluate pre-post changes in stigma within the target populations for the social marketing campaigns ran in Ghana and Kenya carried out as components of the wider Time to Change Global programme.

**Methods:**

Using data collected before and after each campaign in Accra and Nairobi, we investigated pre-post differences in stigma-related outcome measures: mental health-related knowledge (MAKS), mental health-related attitudes (CAMI), and desire for social distance (RIBS), with regression analyses. Other covariates were included in the models to control for differences in participant demographics.

**Results:**

A significant positive change in a stigma related outcome was found at each site. Reported in standard deviation units, desire for social distance from people with mental health problems in Accra was lower after the launch of the campaign, measured as an increase in intended contact (β = 0.29, 95% CI = 0.14 to 0.43, *p* < 0.001). In Nairobi, the stigma related knowledge score was higher in the post campaign sample (β = 0.21, 95% CI = 0.07 to 0.34, *p* = 0.003).

**Conclusion:**

The increase in intended contact in the absence of other changes seen in Ghana, is consistent with the early results for Time to Change England. The estimate for the magnitude of this change is the same as Time to Change England for the general population between 2009 and 19, a very promising result for a short term public mental health campaign. The different results observed between sites may be due to campaign as well as population differences.

**Supplementary Information:**

The online version contains supplementary material available at 10.1186/s12889-021-10966-8.

## Background

Developing strategies for reducing stigma was identified as key priority in the World Health Organisation Mental Health Action Plan 2013–2020 [1]. Structural level stigma and discrimination is apparent from the lack of priority often given to mental health on the part of policymakers, and from the abuses of human rights in psychiatric institutions across countries of all income levels [2]. At the population level, lack of knowledge and stigmatising beliefs have also been identified as a barrier to mental health care [3]. Stigma reduction is therefore essential to both provision and use of mental health care.

Most research on stigma reduction interventions has been conducted in high income countries (HICs) [4], and there is a growing literature on stigma reduction interventions in low- and middle- income countries (LMICs) [5]. Use of social media has the potential to reach large numbers at low cost, however while social marketing using social and/or mass media has been used in LMICs there has been little evaluation. In HICs there is evidence that when combined with other strategies its use is associated with population level changes in stigma related outcomes [6–8].

Time to Change Global is a new anti-stigma programme in LMICs. It developed from Time to Change England, a campaign led by the UK mental health charities Mind and Rethink since 2007 [9]. Funded by the UK Government and Comic Relief, Time to Change Global is delivered in partnership with Christian Blind Mission UK (CBM UK). It was launched in 2019 in Ghana, Nigeria, Kenya, Uganda and India, where there is evidence of high levels of stigma and discrimination towards people with mental health problems [10–12] and all have partner English speaking non-governmental organisations (NGOs) to work alongside.

Its aims are to: improve public health attitudes and behaviour towards people with mental health problems; support those with lived experience to challenge stigma and discrimination in their communities; support NGOs to run local anti-stigma campaigns led by those with lived experience; encourage a culture or shared learning across international boundaries; and create a sustainable global campaign.

In late 2019 and early 2020, Time to Change Global and its partners in Ghana, Nigeria, Kenya and Uganda ran a series of social marketing pilot campaigns in each respective capital city. In the case of Ghana and Kenya, budget was assigned for enhanced evaluation of the pilot campaigns. In Accra, Ghana, the target sociodemographic groups for the social marketing campaign were A, B, C1 and C2, while in Kenya they were A, B, C and D. These were determined using a living standards questionnaire and are further described in the methods section. At both sites the campaign targeted adults aged 18 to 34 years.

### Aim of the study

This study aims to evaluate the social marketing campaigns in Ghana and Kenya with respect to reduction in stigma in the target populations, as measured for the Time to Change England programme. We also investigate whether stigma outcomes vary by demographic groups, as this information can inform the design of subsequent campaigning.

## Methods

### Design

This study used a pre-post design to compare stigma related outcome measures among samples of the target populations. Each campaign ran in the capital cities of Accra and Nairobi, where the urban populations are 2.5 and 4.4 million respectively [13, 14]. Ghana and Kenya have lower-middle income economies according to the World Bank classification for the 2020 fiscal year [15]. Two samples were recruited, the week prior to the campaign launch and the week immediately after the campaign had finished. The samples are residents of each of Greater Accra and Nairobi who are aged 18–34, of social grade ABCD in Nairobi and grades ABC1C2 in Accra, with an equal focus on men and women. These groups are regular users of social media via mobile devices. Smartphone penetration was 21% in Kenya and 24% in Ghana in 2018, but we expect this figure to be higher in cities and amongst our target socioeconomic group [16].

### Intervention

Focus group discussions were held in each city before creative and media strategies were devised to make efficient use of the budget and employ the right mix of digital and traditional channels for maximum impact. This was done in partnership with local NGOs, MEHSOG (Ghana) and Basic Needs Basic Rights (Kenya), and working with marketing agencies, Now Available Africa (Ghana) and Scanad (Kenya).

#### Ghana

The social marketing campaign in Ghana ran from October to December 2019 using primarily Facebook paid media to reach the key target audience for the campaign. It launched with a “teaser” video and was followed by four video format advertisements featuring mental health Champions talking about their experiences of stigma as well as static advertisement formats designed to dispel myths regarding those with mental health problems (www.itcouldbeyou.com.gh). The campaign reach from this channel was 2.7 million, 52.3% more than target. The campaign also made use of more traditional channels; a single billboard poster site was deployed at a high traffic area in central Accra and interviews were aired on two local radio stations where they reached 84,000 listeners. Twitter was also used as added media traction to host the radio shows’ live conversations. The core campaign message was “It Could Be You” following qualitative research indicating that empathy and engagement was triggered by the understanding of the commonality of mental health problems.

#### Kenya

In Kenya, the campaign ran for 8 weeks from January 6th 2020 predominantly across digital channels Facebook, Instagram and Twitter owing to the heavy mobile usage of the audience and the conversational nature of these platforms. Over the full campaign, Facebook and Instagram advertisements reached approximately 1.6 million people (50% of our target audience), each person seeing the ad at least 3 times. Advertisements on twitter were served over 1.3 million times and garnered over 67,000 tweet engagements. “Presenter mentions” were also bought with a local youth-oriented radio station and supplemented by an interview feature with a mental health Champion. The campaign was conducted for four weeks with four shows daily, each show reaching over 1 million people. Initial research suggested that directly encouraging conversation around the subject of mental health and stigma would resonate with the target audience, and hence a campaign theme of “Speak Up” was used (www.speakup.co.ke).

### Data collection

For the pre-post data collection, stratified multi-stage simple random sampling was used. Regions were split into primary sampling units each of which had a quota. Households and individuals within households were then selected at random. Respondents were interviewed face to face using computer assisted personal interviewing (CAPI). In Ghana, pre data were collected in early October 2019 before the campaign launch on 21/10/19. Post data were collected in January 2020. In Nairobi, Pre data were collected in early January 2020 before the campaign launch, and post data collected March 2020.

### Measures

#### Mental health-related knowledge (MAKS)

Mental health-related knowledge was measured by the Mental Health Knowledge Schedule (MAKS) [17]. This comprises six items covering stigma-related mental health knowledge areas: help seeking, recognition, support, employment, treatment, and recovery, and six items that inquire about classification of various conditions as mental illnesses. The total of the first six items score was standardised. A higher standardised MAKS score represents greater knowledge. Overall test-retest reliability of the MAKS is 0·71 (Lin’s concordance statistic) and the overall internal consistency among items is 0·68 (Cronbach’s alpha).

Two additional items to address stigma related knowledge in the target populations were developed by Time to Change Global following market research. These are; Mental health problems are genetic – they are passed on through generations, and; Mental health problems can be caused by a curse. To allow comparison with Time to Change England and other work using the MAKS, we analysed the responses to these questions separately.

#### Mental health-related attitudes (CAMI)

Public attitude towards mental health was assessed using 12 items from the Community Attitudes toward Mental Illness (CAMI) scale [18]. The 12 items were taken from the longer 26-item version, the UK Department of Health Attitudes to Mental Illness questionnaire, which was used in the general population survey ‘Attitudes to Mental Illness’ conducted throughout Time to Change England [19]. This 12-item version was used in the Health Survey for England (HSE) 2014 [20] and as part of the evaluation of the Phase 3 Time to Change England social marketing campaign [7]. Piloting for use in HSE showed that the 12 items span across both factors identified for the 26-item version (Prejudice and Exclusion, and Tolerance and Support for Community Care) and these factors showed good internal consistency of 0.767 and 0.668 (Cronbach’s alpha) [20]. The standardised total score was used; a higher score represents less stigmatising attitudes.

#### Desire for social distance (RIBS)

Desire for social distance, i.e. the level of intended future contact with people with mental health problems, was measured using the Reported and Intended Behaviour Scale (RIBS) [21]. This assesses four intended contact domains: living with, working with, living nearby and continuing a relationship with someone with a mental health problem, which were derived from the Star Social Distance Scale [22]. The standardised total score was used where a higher score indicates less desire for social distance. Overall test-retest reliability of the total RIBS score is 0·75 (Lin’s concordance statistic) and the overall internal consistency of the scale is 0·85 (Cronbach’s alpha).

#### Socio-economic status

Respondent demographics were recorded as part of the interview (see Supplementary 1). Socio-economic status (SES) was calculated using a living standards questionnaire comprising a list of household items or scenarios with assigned score, covering areas such as facilities, housing type, density of local area, lifestyle luxuries and education and occupation of household lead, where the respondent would answer yes or no to each. Respondents were then categorised into one of four groups (AB, C1, C2, D) based on their total score.

#### Familiarity with mental health problems

Knowing someone with a mental health problem or familiarity with mental illness is strongly associated with stigma related knowledge, attitudes and desire for social distance [23]. Consistent with previous Time to Change evaluations, familiarity was measured using the question: Does anyone close to you have or have had some kind of mental health problem? The responses were categorised into three groups; self-familiarity, other and no familiarity. For analysis, self-familiarity was combined with other familiarity due to very low frequencies of those who reported having had a mental health problem.

#### Campaign awareness

Unprompted campaign awareness was assessed for the following types of media; radio, newspapers/magazines, online, TV and social media. Participants who reported seeing a mental health campaign on any of the domains were categorised as being campaign aware while those who responded ‘no’ or ‘don’t know’ were categorised as not campaign aware.

### Statistical analysis

Descriptive statistics for patient demographic and crude outcome scores were calculated and presented by country. Three multiple linear regression models were used to evaluate changes in: (i) public knowledge (MAKS); (ii) public attitudes (CAMI); and (iii) public desire for social distance (RIBS) of mental health problems and two logistic regression models were used to evaluate changes in the proportion of respondents who (negatively) agreed to the additional MAKS items on mental health problems being heredity and a curse.

To evaluate changes before and after the campaign all models included a fixed effect for pre/post the campaign using a categorical dummy variable. Other covariates were included in the models to control for differences in participant demographics: gender (Male, Female), Age (18–25, 26–34), Working status (Student, Working, Not working), Living alone (Yes, No), Living with Children (Yes, No), Religion (Pentecostal/Charismatic, Catholic/Anglican, Presbyterian, Baptist, Methodist, Seventh-Day Adventist (SDA), Other Christians, Islam, No religion, Traditional religion, Other religion), Education (Junior High or lower, Secondary, Post-secondary education, University education), Marriage status (Married, Living with someone in a stable relationship, Single), Socio-economic status (AB, C1,C2,D) and Familiarity with mental health problems (Self or other, None). The demographic variables were chosen based on relationships found in previous literature.

As the linear models used standardised scores of the measures as the dependent variables, the results were interpreted in standard deviation units.

## Results

### Sample demographics at each site

The demographics of participants for each sample are reported in Table 1, which reflect differences in the target populations as well as demographic differences between the settings in terms of variables such as religion. All samples had an equal proportion of male and female respondents, and 81–85% of respondents from both countries did not live alone. Ghana represented a younger sample, 68% of both pre and post samples were aged 18–25 years, whereas 65 and 60% of the Kenya pre and post samples respectively were aged 26–34 years. Kenya had a high proportion of respondents working both pre and post the campaign, 87 and 82% respectively, whereas Ghana had lower rates of working respondents, 60 and 57% of pre and post samples. Level of education amongst Ghana respondents was lower, 16 and 15% of pre and post samples had only primary education or lower, whereas only 3% of Kenyan respondents were in this category. Most Kenyan respondents were categorised as socio-economic group D (57 and 63%), whereas no Ghanaian’s were in this category and the majority were categorised as C1 (56 and 55%), reflecting the different target populations. The most common religion amongst Kenyan respondents was Catholic/Anglican (25 and 28%) and the most common amongst Ghanaian respondents was Other Christians (40%) for the pre sample, and Pentecostal/Charismatic (35%) for the post sample. Almost half of the Kenyan respondents were married (49 and 40%), whereas most Ghanaian respondents were single (78 and 84%).

**Table 1:** Demographics of the Time to Change population, Ghana and Kenya (*n* = 1640)

Variable, n(%)		Ghana (***n*** = 810)		Kenya (***n*** = 830)	
		Pre (***n*** = 408)	Post (***n*** = 402)	Pre (***n*** = 419)	Post (***n*** = 411)
Gender					
Male		206 (50.5)	201 (50.0)	211 (50.4)	210 (51.1)
Female		202 (49.5)	201 (50.0)	208 (49.6)	201 (48.9)
Age					
18–25		276 (67.6)	272 (67.7)	144 (34.4)	164 (39.9)
26–34		132 (32.4)	130 (32.3)	275 (65.6)	247 (60.1)
Employment status					
Working		247 (60.5)	228 (56.7)	365 (87.1)	338 (82.2)
Student		117 (28.7)	141 (35.1)	41 (9.8)	53 (12.9)
Not working		44 (10.8)	33 (8.2)	13 (3.1)	20 (4.9)
Level of education					
Junior High or lower		66 (16.2)	61 (15.3)	11 (2.6)	11 (2.7)
Secondary		227 (55.8)	188 (47.1)	160 (38.2)	158 (38.4)
Post secondary education		75 (18.4)	84 (21.1)	175 (41.8)	179 (43.6)
University education		39 (9.6)	66 (16.5)	73 (17.4)	63 (15.3)
Religious denomination					
Catholic/Anglican		18 (4.4)	38 (9.5)	105 (25.1)	115 (28.0)
Presbyterian, Baptist, Methodist		60 (14.7)	68 (16.9)	90 (21.5)	75 (18.2)
Seventh Day Adventist (SDA)		9 (2.2)	7 (1.7)	56 (13.4)	46 (11.2)
Pentecostal/Charismatic		121 (29.7)	141 (35.1)	84 (20.1)	85 (20.7)
Other Christians		165 (40.4)	67 (16.7)	52 (12.4)	59 (14.4)
Islam		30 (7.4)	63 (15.7)	14 (3.3)	14 (3.4)
No religion		4 (1.0)	9 (2.2)	16 (3.8)	14 (3.4)
Other religion		1 (0.2)	9 (2.2)	1 (0.2)	0 (0.0)
Marital status					
Married		50 (12.3)	44 (10.9)	204 (48.7)	163 (39.7)
Living with someone in a stable relationship		42 (10.3)	22 (5.5)	17 (4.1)	20 (4.9)
Single		316 (77.5)	336 (83.6)	198 (47.3)	228 (55.5)
Living alone					
Living with others		338 (82.8)	341 (84.8)	338 (80.7)	339 (82.5)
Living Alone		70 (17.2)	61 (15.2)	81 (19.3)	72 (17.5)
Living with children					
No children		206 (50.5)	192 (47.8)	123 (29.4)	133 (32.4)
Living with children		202 (49.5)	210 (52.2)	296 (70.6)	278 (67.6)
Socio-economic class					
AB		31 (7.6)	31 (7.7)	12 (2.9)	11 (2.7)
C1		227 (55.6)	221 (55.0)	31 (7.4)	13 (3.2)
C2		150 (36.8)	150 (37.3)	136 (32.5)	128 (31.1)
D		0 (0.0)	0 (0.0)	240 (57.3)	259 (63.0)
Closest person with a mental illness					
Self		2 (0.5)	0 (0.0)	8 (1.9)	8 (1.9)
Other		176 (43.1)	84 (20.9)	137 (32.7)	150 (36.5)
No-one known		230 (56.4)	318 (79.1)	274 (65.4)	253 (61.6)
Campaign aware	Yes	263 (64.5)	267 (66.4)	125 (29.8)	242 (58.9)
	No	145 (35.5)	135 (33.6)	294 (70.2)	169 (41.1)

In Ghana, overall unprompted awareness of a mental health campaign was high both before (64.5%) and after (66.4%) the campaign, whereas in Kenya, awareness was 29.8% before and 58.9% after the campaign.

### Knowledge of classification of conditions as mental health problems

The percentage of participants who agree to the classification of six conditions as mental health problems (MAKS part B) split by campaign sample and country are shown in Table S2. For both Ghanaian and Kenyan participants, correct responses are high (78–95%), whereas, an incorrect response regarding grief is high in the Ghana sample (78–81%) but lower in the Kenya sample (60–69%). Additionally, an incorrect response regarding stress is high in the Kenya sample (86–87%) but lower in the Ghana sample (69–74%).

### Changes in stigma related outcome measures

Raw outcome data are presented in online supplementary material S2-S4.

### Ghana

Table 2 shows the results from the regression analyses to assess pre-post changes and factors associated with mental health-related knowledge (MAKS plus two additional items), attitudes (CAMI) and behaviour (RIBS IB) in Ghana.

**Table 2:** Regression analyses to assess pre/post changes in mental health-related knowledge, attitudes and behaviour in Ghana (*n* = 806)

Predictors	Knowledge: standardised MAKS score (***n*** = 806)		Knowledge: MAKS heredity item (***n*** = 806)		Knowledge: MAKS curse item (***n*** = 806)		Attitudes: standardised CAMI score (***n*** = 806)		Intended behaviour: standardised RIBS IB score (***n*** = 806)	
	Standardised effect size (95% CI)	*P* value	Standardised effect size (95% CI)	*P* value	Standardised effect size (95% CI)	*P* value	Standardised effect size (95% CI)	*P* value	Standardised effect size (95% CI)	*P* value
Stage										
Post	−0.15 (− 0.30,0.00)	0.050	0.88 (0.65, 1.21)	0.432	0.56 (0.38, 0.82)	0.003	0.11 (− 0.04, 0.27)	0.134	0.29 (0.14, 0.43)	< 0.001
Pre (ref)	–	–	–	–	–	–	–	–	–	–
Gender										
F	−0.03 (− 0.17, 0.11)	0.699	1.26 (0.94, 1.71)	0.128	1.20 (0.83, 1.73)	0.331	−0.02 (− 0.16, 0.13)	0.801	− 0.10 (− 0.24, 0.04)	0.156
M (ref)	–	–	–	–	–	–	–	–	–	–
Age										
18–25	−0.29 (− 0.46, − 0.12)	0.001	1.10 (0.76, 1.59)	0.623	0.79 (0.51, 1.24)	0.305	0.04 (−0.14, 0.21)	0.682	−0.09 (− 0.26, 0.08)	0.290
26–34 (ref)	–	–	–	–	–	–	–	–	–	–
Working										
Student	0.28 (0.11, 0.45)	0.002	0.99 (0.69, 1.43)	0.964	1.23 (0.79, 1.91)	0.357	0.00 (− 0.18, 0.18)	0.999	−0.12 (− 0.29, 0.05)	0.172
Not working	0.34 (0.08, 0.59)	0.009	1.14 (0.67, 1.95)	0.626	1.39 (0.70, 2.76)	0.346	−0.02 (− 0.28, 0.23)	0.871	0.15 (− 0.10, 0.39)	0.235
Working (ref)	–	–	–	–	–	–	–	–	–	–
Living Alone										
Yes	−0.10 (− 0.31, 0.11)	0.353	2.16 (1.37, 3.41)	0.001	1.08 (0.64, 1.83)	0.773	0.19 (−0.02, 0.40)	0.079	0.09 (−0.11, 0.29)	0.381
No (ref)	–	–	–	–	–	–	–	–	–	–
Living with children										
Yes	−0.03 (− 0.18, 0.13)	0.743	1.19 (0.86, 1.64)	0.288	1.09 (0.74, 1.61)	0.659	−0.16 (− 0.31, 0.00)	0.048	0.04 (− 0.11, 0.19)	0.643
No (ref)	–	–	–	–	–	–	–	–	–	–
Religion										
Catholic/Anglican	0.13 (−0.16, 0.41)	0.387	1.26 (0.69, 2.29)	0.450	1.23 (0.58, 2.58)	0.591	0.20 (−0.09, 0.49)	0.184	0.26 (−0.02, 0.55)	0.066
Presbyterian, Baptist, Methodist	0.11 (− 0.10, 0.32)	0.301	1.19 (0.77, 1.84)	0.434	0.98 (0.58, 1.67)	0.939	−0.14 (− 0.35, 0.07)	0.190	0.23 (0.03, 0.44)	0.027
Seventh-Day Adventist (SDA)	−0.16 (− 0.67, 0.34)	0.528	1.12 (0.39, 3.20)	0.835	1.68 (0.36, 7.88)	0.513	0.05 (−0.46, 0.56)	0.856	−0.21 (− 0.71, 0.28)	0.394
Other Christians	0.09 (−0.09, 0.27)	0.312	1.53 (1.04, 2.24)	0.029	0.80 (0.50, 1.27)	0.343	−0.07 (− 0.25, 0.12)	0.479	0.31 (0.13, 0.49)	0.001
Islam	0.12 (−0.12, 0.35)	0.337	1.81 (1.08, 3.04)	0.023	1.10 (0.59, 2.06)	0.766	−0.09 (− 0.33, 0.15)	0.481	0.10 (− 0.13, 0.33)	0.409
No religion	−0.24 (− 0.80, 0.31)	0.393	0.43 (0.13, 1.48)	0.183	0.32 (0.10, 1.04)	0.058	−0.01 (− 0.57, 0.55)	0.973	0.54 (− 0.00, 1.08)	0.051
Other religion	0.13 (−0.50, 0.76)	0.682	1.06 (0.29, 3.92)	0.933	1.43 (0.28, 7.17)	0.666	−0.23 (− 0.86, 0.41)	0.485	0.07 (− 0.55, 0.68)	0.833
Pentecostal/Charismatic (ref)	–	–	–	–	–	–	–	–	–	–
Education										
Junior High or lower	0.02 (−0.27, 0.31)	0.894	1.29 (0.70, 2.40)	0.414	1.58 (0.74, 3.39)	0.240	−0.13 (− 0.42, 0.17)	0.395	− 0.08 (− 0.36, 0.20)	0.577
Secondary	− 0.19 (− 0.43, 0.04)	0.101	1.03 (0.63, 1.67)	0.919	1.20 (0.68, 2.14)	0.530	−0.15 (− 0.38, 0.09)	0.214	− 0.24 (− 0.46, − 0.01)	0.040
Post secondary education	−0.07 (− 0.32, 0.18)	0.598	0.78 (0.46, 1.32)	0.365	0.73 (0.40, 1.33)	0.305	0.03 (−0.22, 0.29)	0.796	−0.14 (− 0.39, 0.10)	0.254
University education (ref)	–	–	–	–	–	–	–	–	–	–
Marriage status										
Married	0.001 (−0.25, 0.25)	0.995	1.39 (0.81, 2.37)	0.231	1.09 (0.56, 2.11)	0.802	0.02 (−0.23, 0.27)	0.869	0.04 (−0.21, 0.28)	0.769
Cohabiting	−0.24 (− 0.51, 0.02)	0.073	0.89 (0.51, 1.56)	0.693	0.52 (0.28, 0.98)	0.044	0.00 (−0.26, 0.27)	0.974	−.011 (− 0.37, 0.14)	0.380
Single (ref)	–	–	–	–	–	–	–	–	–	–
Socio-economic status										
AB	−0.03 (− 0.32, 0.26)	0.842	1.12 (0.61, 2.07)	0.712	0.76 (0.37, 1.59)	0.471	0.06 (−0.24, 0.36)	0.699	0.48 (0.20, 0.77)	0.001
C1	−0.09 (− 0.24, 0.07)	0.253	1.19 (0.85, 1.66)	0.307	0.82 (0.54, 1.24)	0.345	0.11 (− 0.05, 0.27)	0.183	0.21 (0.06, 0.37)	0.006
C2 (ref)	–	–	–	–	–	–	–	–	–	–
Familiarity with mental health problems										
Self or other	0.20 (0.04, 0.35)	0.012	1.61 (1.16, 2.23)	0.004	1.54 (1.02, 2.33)	0.041	0.12 (−0.04, 0.27)	0.136	0.33 (0.18, 0.48)	< 0.001
None (ref)	–	–	–	–	–	–	–	–	–	–

The post Ghana campaign sample had a slightly lower MAKS score then those who responded before the campaign (pre), and this change was borderline significant at the 5% level (β = − 0.15, 95% CI = -0.30 to 0.00, *p* = 0.05). The results also show the younger group (18–25 years) had lower levels of stigma related knowledge (lower total MAKS score) compared to the older group (26–34 years) (β = − 0.30, 95% CI = -0.46 to − 0.12, *p* = 0.001), and students (β = 0.28, 95% CI = 0.10 to 0.45, *p* = 0.002) or non-working respondents (β = 0.34, 95% CI = 0.08 to 0.59, *p* = 0.009) had better stigma related knowledge (higher MAKS score) compared to those who were working. The model also shows those with familiarity with mental health problems, via themselves or others, had better stigma related knowledge than those with no familiarity (β = 0.19, 95% CI = 0.04 to 0.35, *p* = 0.012).

Table 2 also shows the results from the separate logistic regression models to assess changes in proportions who agreed with the two additional stigma related knowledge statements, that mental illness is caused by genetics and by a curse. There was no statistically significant difference between the pre and post samples in agreement with the statement that mental-health problems are genetic. Those living alone had 2.16 times higher odds of agreeing to the heredity statement than those who were not (OR = 2.16, 95% CI = 1.37 to 3.41, *p* = 0.001). Other Christians (OR = 1.53, 95% CI = 1.04 to 2.24, *p* = 0.029) and Islam/Muslim (OR = 1.81, 95% CI = 1.08 to 3.04, *p* = 0.023) respondents also had 1.53 and 1.81, respectively, higher odds of agreeing to the statement that mental-health problems are genetic, than those respondents who were of Pentecostal or Charismatic religion. Respondents who had familiarity with mental health problems, either through oneself or others, had 1.61 times higher odds of agreeing to the statement that mental-health problems are genetic, than those respondents with no familiarity (OR = 1.61, 95% CI = 1.16 to 2.23, *p* = 0.004).

The post campaign sample had 0.56 times lower odds of agreeing to the statement that mental health problems can be caused by a curse, compared to the pre sample (OR = 0.56, 95% CI = 0.38 to 0.82, *p* = 0.003). Respondents who were living with someone in a stable relationship had 0.53 times lower odds of agreeing to the statement that mental health problems can be caused by a curse, than respondents who were single (OR = 0.53, 95% CI = 0.28 to 0.98, *p* = 0.044). Respondents who had familiarity with mental health problems, either through oneself or others, had 1.54 times higher odds of agreeing to the statement that mental-health problems can be caused by a curse, than those respondents with no familiarity (OR = 1.54, 95% CI = 1.02 to 2.33, *p* = 0.041).

Table 2 also shows no pre-post change was found in mental health related attitudes. The only demographic factor associated with the attitudes score was whether respondents were living with children; those who did tend to express more negative attitudes (β = − 0.16, 95% CI = -0.31 to − 0.001, *p* = 0.048).

The post Ghana campaign sample had a higher score on the RIBS scale (less desire for social distance) than respondents interviewed before the campaign (β = 0.29, 95% CI = 0.14 to 0.43, *p* < 0.001). The results also show those respondents who were of Presbyterian, Baptist or Methodist (β = 0.23, 95% CI = 0.03 to 0.44, *p* = 0.027) or other Christian religions (β = 0.31, 95% CI = 0.13 to 0.48, *p* = 0.001) had less desire for social distance than Pentecostal/Charismatic respondents. Those who were from higher economic groups, AB (β = 0.48, 95% CI = 0.20 to 0.77, *p* = 0.001) and C1(β = 0.21, 95% CI = 0.06 to 0.37, *p* = 0.006), also had less desire for social distance than those from socioeconomic group C2, and those who had familiarity with mental health problems through either themselves or others had less desire for social distance than those who had no familiarity (β = 0.33, 95% CI = 0.18 to 0.48, *p* < 0.001). Those whose highest level of education was Secondary had more desire for social distance than those who had University education (β = − 0.24, 95% CI = -0.46 to − 0.01, *p* = 0.004).

### Kenya

Table 3 shows the results from the regression analyses to assess pre-post changes and factors associated with mental health-related knowledge (MAKS plus two additional items), attitudes (CAMI) and behaviour (RIBS IB) in Kenya.

**Table 3:** Regression analyses to assess pre/post changes in mental health-related knowledge, attitudes and behaviour in Kenya (*n* = 829)

Predictors	Knowledge: standardised MAKS score (***n*** = 829)		Knowledge: MAKS heredity item (***n*** = 829)		Knowledge: MAKS curse item (***n*** = 829)		Attitudes: standardised CAMI score (***n*** = 829)		Intended behaviour: standardised RIBS IB score (***n*** = 829)	
	Standardised effect size (95% CI)	*P* value	Standardised effect size (95% CI)	*P* value	Standardised effect size (95% CI)	*P* value	Standardised effect size (95% CI)	*P* value	Standardised effect size (95% CI)	*P* value
Stage										
Post	0.21 (0.07, 0.34)	0.003	1.11 (0.83, 1.49)	0.491	0.67 (0.49, 0.91)	0.010	0.04 (− 0.10, 0.17)	0.583	− 0.04 (− 0.17, 0.08)	0.511
Pre (ref)	–	–	–	–	–	–	–	–	–	–
Gender										
F	−0.09 (− 0.23, 0.06)	0.247	0.89 (0.64, 1.23)	0.475	0.67 (0.48, 0.93)	0.018	−0.03 (− 0.18, 0.11)	0.665	− 0.11 (− 0.24, 0.03)	0.122
M (ref)	–	–	–	–	–	–	–	–	–	–
Age										
18–25	0.17 (− 0.001, 0.34)	0.051	0.83 (0.57, 1.21)	0.343	0.83 (0.56, 1.24)	0.366	0.03 (−0.14, 0.20)	0.758	0.07 (− 0.09, 0.23)	0.363
26–34 (ref)	–	–	–	–	–	–	–	–	–	–
Working										
Student	−0.11 (− 0.36, 0.13)	0.366	1.35 (0.79, 2.32)	0.274	1.14 (0.65, 2.00)	0.652	−0.21 (− 0.45, 0.04)	0.094	− 0.17 (− 0.40, 0.05)	0.133
Not working	0.11 (− 0.24, 0.47)	0.542	0.39 (0.15, 0.99)	0.048	0.55 (0.23, 1.34)	0.190	−0.01 (− 0.36, 0.35)	0.976	0.19 (− 0.14, 0.52)	0.252
Working (ref)	–	–	–	–	–	–	–	–	–	–
Living Alone										
Yes	0.01 (− 0.24, 0.26)	0.934	1.28 (0.72, 2.28)	0.407	1.04 (0.57, 1.90)	0.907	−0.17 (− 0.42, 0.08)	0.193	− 0.19 (− 0.42, 0.05)	0.118
No (ref)	–	–	–	–	–	–	–	–	–	–
Living with children										
Yes	0.12 (−0.11, 0.35)	0.290	1.35 (0.80, 2.29)	0.261	1.53 (0.89, 2.64)	0.127	−0.20 (− 0.43, 0.03)	0.090	− 0.03 (− 0.24, 0.18)	0.767
No (ref)	–	–	–	–	–	–	–	–	–	–
Religion										
Catholic/Anglican	0.08 (−0.11, 0.28)	0.406	0.88 (0.57, 1.36)	0.576	0.93 (0.59, 1.47)	0.754	0.24 (0.04, 0.44)	0.017	0.25 (0.06, 0.43)	0.008
Presbyterian, Baptist, Methodist	0.24 (0.02, 0.45)	0.029	0.77 (0.48, 1.22)	0.269	1.16 (0.72, 1.87)	0.549	0.33 (0.12, 0.55)	0.002	0.40 (0.21, 0.60)	< 0.001
Seventh-Day Adventist (SDA)	0.03 (− 0.21, 0.28)	0.779	1.04 (0.62, 1.76)	0.871	1.10 (0.64, 1.91)	0.726	0.11 (−0.13, 0.36)	0.352	0.16 (− 0.07, 0.38)	0.166
Other Christians	0.08 (− 0.16, 0.32)	0.516	1.12 (0.67, 1.86)	0.669	1.96 (1.17, 3.29)	0.011	0.14 (−0.09, 0.38)	0.232	0.42 (0.20, 0.64)	< 0.001
Islam	0.79 (0.39, 1.18)	< 0.001	1.41 (0.60, 3.33)	0.430	4.44 (1.84, 10.75)	0.001	−0.13 (−0.52, 0.27)	0.527	0.14 (−0.22, 0.51)	0.445
No religion	0.68 (0.29, 1.06)	0.001	0.76 (0.32, 1.83)	0.543	6.04 (2.44, 14.94)	< 0.001	0.74 (0.35, 1.12)	< 0.001	0.95 (0.59, 1.31)	< 0.001
Other religion	0.62 (−0.36, 1.60)	0.216	1.82 (0.23, 14.31)	0.568	2.25 (0.28, 17.92)	0.443	−0.48 (−1.45, 0.50)	0.337	−0.09 (−1.00, 0.82)	0.853
Pentecostal/Charismatic (ref)	–	–	–	–	–	–	–	–	–	–
Education										
Junior High or lower	−0.31 (− 0.77, 0.16)	0.199	1.98 (0.71, 5.53)	0.193	4.18 (1.46, 11.91)	0.007	−0.29 (− 0.75, 0.18)	0.226	− 0.16 (− 0.60, 0.27)	0.463
Secondary	0.09 (− 0.14, 0.32)	0.455	2.19 (1.26, 3.80)	0.005	2.84 (1.57, 5.14)	0.001	0.00 (−0.23, 0.24)	0.968	0.28 (0.06, 0.49)	0.012
Post secondary education	−0.08 (− 0.29, 0.13)	0.447	2.78 (1.67, 4.63)	< 0.001	3.04 (1.74, 5.30)	< 0.001	−0.16 (− 0.37, 0.05)	0.145	0.00 (− 0.20, 0.19)	0.966
University education (ref)	–	–	–	–	–	–	–	–	–	–
Marriage status										
Married	0.04 (−0.14, 0.21)	0.683	1.40 (0.96, 2.04)	0.082	0.97 (0.66, 1.44)	0.887	0.07 (−0.10, 0.24)	0.433	−0.01 (− 0.17, 0.15)	0.860
Cohabiting	0.26 (−0.08, 0.60)	0.132	0.77 (0.35, 1.69)	0.518	0.99 (0.45, 2.20)	0.987	0.08 (−0.26, 0.42)	0.643	−0.10 (− 0.42, 0.22)	0.530
Single (ref)	–	–	–	–	–	–	–	–	–	–
Socio-economic status										
AB	0.16 (−0.27, 0.58)	0.467	1.36 (0.55, 3.38)	0.508	1.10 (0.43, 2.84)	0.839	−0.36 (− 0.78, 0.06)	0.092	0.12 (− 0.27, 0.51)	0.551
C1	0.12 (−0.20, 0.44)	0.451	1.24 (0.61, 2.51)	0.556	0.71 (0.33, 1.53)	0.381	−0.38 (− 0.70, − 0.07)	0.018	−0.16 (− 0.45, 0.14)	0.303
C2	0.12 (−0.04, 0.29)	0.129	0.63 (0.44, 0.90)	0.011	0.70 (0.48, 1.01)	0.055	−0.06 (− 0.22, 0.10)	0.479	− 0.01 (− 0.16, 0.14)	0.879
D (ref)	–	–	–	–	–	–	–	–	–	–
Familiarity with mental health problems										
Self or other	0.24 (0.10, 0.38)	0.001	0.99 (0.73, 1.35)	0.967	0.93 (0.67, 1.28)	0.650	0.40 (0.26, 0.54)	< 0.001	0.78 (0.64, 0.91)	< 0.001
None (ref)	–	–	–	–	–	–	–	–	–	–

The post Kenya campaign sample had a higher total MAKS score (higher stigma related knowledge) compared to the pre campaign sample (β = 0.21, 95% CI = 0.07 to 0.34, *p* = 0.003). Respondents who were of Presbyterian, Baptist, Methodist (β = 0.24, 95% CI = 0.02 to 0.45, *p* = 0.029) or Islam (β = 0.79, 95% CI = 0.39 to 1.18, *p* < 0.001) or no religion (β = 0.68, 95% CI = 0.29 to 1.06, *p* = 0.001) had a higher total MAKS score than Pentecostal/Charismatic respondents. Those with familiarity with mental health problems, via themselves or others, had higher stigma related knowledge than those with no familiarity (β = 0.24, 95% CI = 0.10 to 0.38, *p* = 0.001).

Table 3 also shows the results from the separate logistic regression models to assess changes in proportions who agreed with the two additional stigma related knowledge statements, that mental illness is caused by genetics and by a curse. There was no statistically significant difference between the pre and post campaign samples in agreement with the statement that mental-health problems are genetic. Those respondents that were not working had 0.39 times lower odds of agreeing to the heredity item than those respondents who were working (OR = 0.39, 95% CI = 0.15 to 0.99, *p* = 0.048). Respondents that had secondary education (OR = 2.19, 95% CI = 1.26 to 3.80, *p* = 0.005) or post-secondary education (OR = 2.78, 95% CI = 1.67 to 4.63, *p* < 0.001) as their highest education level had 2.19 and 2.78 times higher odds respectively, of agreeing to the statement than respondents who had university education. Respondents belonging to socioeconomic group C2 had 0.63 times lower odds of agreeing than respondents of socioeconomic group D (OR = 0.63, 95% CI = 0.44 to 0.90, *p* = 0.011).

The post campaign sample had 0.67 times lower odds of agreeing to the statement that mental health problems can be caused by a curse, as compared to the pre campaign sample (OR = 0.67, 95% CI = 0.49 to 0.91, *p* = 0.010). Female respondents also had 0.67 times lower odds of agreeing to the statement than male respondents (OR = 0.67, 95% CI = 0.48 to 0.93, *p* = 0.018). Respondents who were other Christians (OR = 1.96, 95% CI = 1.17 to 3.29, *p* = 0.011), Muslim (OR = 4.44, 95% CI = 1.84 to 10.75, *p* = 0.001) or practiced no religion (OR = 6.04, 95% CI = 2.44 to 14.94, *p* < 0.001) had significantly higher odds of agreeing than Pentecostal/Charismatic respondents. Respondents of lower education categories, Junior high (OR = 4.18, 95% CI = 1.46 to 11.91, *p* = 0.007), secondary (OR = 2.84, 95% CI = 1.57 to 5.14, *p* = 0.001) or post-secondary (OR = 3.04, 95% CI = 1.74 to 5.30, *p* < 0.001), also had significantly higher odds of agreeing to the statement that mental health problems can be caused by a curse than respondents with university education.

The results in Table 3 also show no change in mental health related attitudes between respondents before and after the campaign. Respondents who practised no religion (β = 0.74, 95% CI = 0.35 to 1.12, *p* < 0.001), Presbyterian, Baptist or Methodist (β = 0.33, 95% CI = 0.12 to 0.55, *p* = 0.002), or Catholic/Anglican (β = 0.24, 95% CI = 0.04 to 0.44, *p* = 0.017) had more positive attitudes than those who were of Pentecostal/Charismatic religion. Respondents with familiarity, via either themselves or others, had a higher CAMI total score (more positive attitudes) than those respondents with no familiarity (β = 0.40, 95% CI = 0.26 to 0.54, *p* < 0.001). The results also show that respondents of socioeconomic group C1 had more negative attitudes than those of socioeconomic group DE (β = − 0.38, 95% CI = -0.70 to − 0.07, *p* = 0.018).

In Kenya there was no difference between pre and post campaign samples in desire for social distance. Those respondents who were of Presbyterian, Baptist or Methodist (β = 0.40, 95% CI = 0.21 to 0.60, *p* < 0.001), Catholic or Anglican (β = 0.25, 95% CI = 0.06 to 0.43, *p* = 0.008), other Christian religions (β = 0.42, 95% CI = 0.20 to 0.64, *p* < 0.001) or no religion (β = 0.95, 95% CI = 0.59 to 1.31, *p* < 0.001) had less desire for social distance than Pentecostal/Charismatic respondents. Those whose highest form of education was secondary also had less desire for social distance than those who had University education (β = 0.28, 95% CI = 0.06 to 0.49, *p* = 0.012). Respondents who had familiarity with mental health problems through either themselves or others had less desire for social distance than those who had no familiarity (β = 0.78, 95% CI = 0.64 to 0.91, *p* < 0.001).

## Discussion

Whilst some results showed no change, a significant positive change in a stigma related outcome was found in each site. Desire for social distance from people with mental health problems in Accra reduced after the campaign. This positive change in intended behaviour in the absence of improvements in attitudes or stigma related knowledge is consistent with the early results for the Attitudes to Mental Illness Survey of the general population carried out to evaluate Time to Change England [24]. The estimate for the magnitude of this change is the same as that over the course of Time to Change England for the general population between 2009 and 19 [19], although the smaller sample size in Time to Change Global means it is less precise. This promising result for a short term public mental health campaign may reflect the greater scope for change in Ghana, where the baseline scores are generally lower than for England, as well as a readiness to engage with the campaign.

In Kenya we observed a different pattern, in that the stigma related knowledge score was higher in the post campaign sample while there was no difference for attitudes or intended behaviour. This may reflect differences in the campaigns as well as population differences.

In both sites there were reduced levels of agreement with the additional item on whether mental health problems are caused by a curse, but not with the heredity item, in the post sample.

In comparison with surveys in HICs, differences in stigma measures by demographic factors such as gender and socioeconomic status were less pronounced [8, 23, 25]. However, religion was associated with the curse item and desire for social distance in both sites, for RIBS in Ghana, and with the total MAKS, CAMI and RIBS scores for Kenya. The most consistent finding was that having no religion was associated with more positive outcomes, as compared to Pentecostal or charismatic faith.

Consistent with surveys elsewhere [23], familiarity with mental illness through personal experience or a relationship was associated with all outcomes in Kenya. In Ghana this was the case for knowledge and intended behaviour but not for attitudes, and the relationships were weaker. Notably, the odds of Ghanaian participants agreeing with the additional items to assess belief on whether mental health problems are genetic and caused by a curse were higher in the presence of familiarity.

### Strengths and limitations of the study

To our knowledge this is the first evaluation of a social marketing campaign using social and mass media in two countries with lower-middle income economies.

This study uses well established stigma related outcome measures which have been used in numerous anti-stigma evaluations globally. The questions were tested in the market research phase for acceptability and relevance. At the same time, additional items were used to capture relevant knowledge domains and examined separately.

Collection of demographic measures meant observed confounders could be adjusted for, however there may be unobserved confounders that we were unable to adjust for. Multiple religious groups were examined as there may be important differences not just among the major religions but also between denominations, however this also meant low frequencies in some groups. Stratified sampling ensured pre and post samples within each country were comparable and results can be generalised to the population.

A limitation of this study is our inability to attribute the changes observed, or to estimate the proportion of the changes observed, to the Time to Change Global programme. Other events may contribute to the changes observed; however the short campaign periods reduce the probability of this. The pre-post evaluation design reflects the impossibility of selecting a control group within each country when using social media to deliver a campaign, as this cannot be limited geographically. Instead, the pre and post sample sizes were maximised within the campaign budget and are larger than those used to evaluate the Time to Change England social marketing campaign [7].

The measures of campaign awareness for Ghana should be treated with caution. This was high both before and after the Time to Change Global campaign suggesting conflation with publicity of World Mental Health Day which was 10 days before the Ghana campaign launch. In Kenya, an increase in campaign awareness was observed after the campaign which was more in line with what we expect.

A further limitation is that the survey does not cover specific diagnoses. The public concept of what constitutes a mental illness may vary person to person or more generally, country to country. Results from this evaluation did highlight the high proportions of Ghanaian respondents who agreed grief was a mental illness, and Kenyan respondents who agreed stress was a mental illness. Levels of stigma may vary with diagnosis and we do not know which conditions participants have in mind when giving their responses.

It is also important to note the evaluation had a relatively short follow-up period which could mean a short-term only impact has been detected [26]. Furthermore, these campaigns were targeted to capital cities and therefore testing of country wide campaigns are needed. Smartphone and internet access would also need to be considered particularly when expanding to rural areas.

### Implications

Our results have implications both for anti-stigma campaign delivery and content. In terms of delivery, use of social and mass media represents a promising strategy in countries of any income in which smartphone usage is widespread at least within the target groups. Local and current research on the social media platforms used by the target groups is needed so that campaign funds can be directed efficiently. In terms of content, the promotion of familiarity through greater recognition and/or disclosure within relationships as has been done in HICs may also be effective in LMICs. However, caution is needed given our results suggest this strategy may be more effective in Kenya than in Ghana, and given the potential adverse consequences of disclosure to individuals with a mental health problem.

In addition to the different campaign content developed for each site, it appears that differences based on religion may require more targeted work within sites. This does not only apply to countries where the majority of the population have a religious affiliation, but to largely secular countries with minority groups for whom religion and mental health are related in important ways [27]. This implies further work is needed to better understand the role of religion and target anti-stigma work in a way that is neither counterproductive nor leads to widening of pre-existing differences in stigma by religious affiliation. A recent systematic review on mental health promotion and stigma reduction in Black faith communities emphasised important features of such work if it is to be effective, notably partnership working and co-production of content [28].

There is a risk that a short-term campaign will have short term results [26]. While a longer follow up would help to determine this, the implication for organisations delivering such campaigns is to plan and evaluate much longer term programmes, such have been running in several HICs [6, 29, 30].

## Supplementary Information

**Additional file 1: Supplementary 1.** Understanding Mental Health Stigmatization and Discrimination Questionnaire September, 2019

**Additional file 2: Table S2.** Percentage (%) of participants who strongly or slightly agree to the MAKS Items and additional knowledge items by Site and Pre vs. Post. **Table S3.** Percentage (%) of participants who strongly or slightly agree to the CAMI Items by Site and Pre vs. Post. **Table S4.** Percentage (%) of participants who strongly or slightly agree to the RIBS IB Items by Site and Pre vs. Post.

## Data Availability

Data are not yet available as this is part of an ongoing programme. At the end of the study they may become available on reasonable request to the senior author (Claire Henderson), subject to approval from the funders.

## Abbreviations

MAKS: Mental Health Knowledge Schedule
CAMI: Community Attitudes toward Mental Illness Scale
RIBS: Reported and Intended Behaviour Scale
SES: Socio-economic status
LMICs: Low- and middle-income countries
HICs: High income countries
NGO: Non-governmental organisation

## References

[1] World Health Organization W. Mental health action plan 2013-2020. 2013.

[2] Lewis O, Campbell A (2017). Violence and abuse against people with disabilities: a comparison of the approaches of the European court of human rights and the United Nations Committee on the rights of persons with disabilities. Int J Law Psychiatry.

[3] Eaton J, McCay L, Semrau M, Chatterjee S, Baingana F, Araya R, Ntulo C, Thornicroft G, Saxena S (2011). Scale up of services for mental health in low-income and middle-income countries. Lancet.

[4] Thornicroft G, Mehta N, Clement S, Evans-Lacko S, Doherty M, Rose D, Koschorke M, Shidhaye R, O'Reilly C, Henderson C (2016). Evidence for effective interventions to reduce mental-health-related stigma and discrimination. Lancet.

[5] Kemp CG, Jarrett BA, Kwon C-S, Song L, Jetté N, Sapag JC, Bass J, Murray L, Rao D, Baral S (2019). Implementation science and stigma reduction interventions in low-and middle-income countries: a systematic review. BMC Med.

[6] Jorm AF, Jorm AF, Christensen H, Griffiths KM, Jorm AF, Christensen H, Griffiths KM (2005). The impact of beyondblue: the national depression initiative on the Australian public's recognition of depression and beliefs about treatments. Aust N Z J Psychiatry.

[7] González-Sanguino C, Potts LC, Milenova M, Henderson C (2019). Time to Change’s social marketing campaign for a new target population: results from 2017 to 2019. BMC Psychiatry.

[8] Hansson L, Stjernswärd S, Svensson B (2016). Changes in attitudes, intended behaviour, and mental health literacy in the Swedish population 2009–2014: an evaluation of a national antistigma programme. Acta Psychiatr Scand.

[9] Henderson C, Thornicroft G (2009). Stigma and discrimination in mental illness: time to change. Lancet.

[10] Maulik PK, Devarapalli S, Kallakuri S, Tripathi AP, Koschorke M, Thornicroft G (2019). Longitudinal assessment of an anti-stigma campaign related to common mental disorders in rural India. Br J Psychiatry.

[11] Mutiso VN, Musyimi CW, Nayak SS, Musau AM, Rebello T, Nandoya E, Tele AK, Pike K, Ndetei DM (2017). Stigma-related mental health knowledge and attitudes among primary health workers and community health volunteers in rural Kenya. Int J Soc Psychiatry.

[12] Barke A, Nyarko S, Klecha D (2011). The stigma of mental illness in southern Ghana: attitudes of the urban population and patients’ views. Soc Psychiatry Psychiatr Epidemiol.

[13] Statistics KNBo. 2019 Kenya Population and Housing Census Volume I: Population by County and Sub-County 2019 [Available from: https://www.knbs.or.ke/?wpdmpro=2019-kenya-population-and-housing-census-volume-i-population-by-county-and-sub-county.

[14] Agency. TWFCI. The World Factbook - Ghana 2013 [Available from: https://www.cia.gov/the-world-factbook/countries/ghana/.

[15] World Bank Country and Lending Groups [Available from: https://datahelpdesk.worldbank.org/knowledgebase/articles/906519-world-bank-country-and-lending-groups.

[16] Newzoo. Newzoo Global Mobile Market Report 2018 - Light Version 2018 [Available from: https://newzoo.com/insights/trend-reports/newzoo-global-mobile-market-report-2018-light-version/.

[17] Evans-Lacko S, Little K, Meltzer H, Rose D, Rhydderch D, Henderson C, Thornicroft G (2010). Development and psychometric properties of the mental health knowledge schedule. Can J Psychiatry.

[18] Taylor SM, Dear MJ (1981). Scaling community attitudes toward the mentally ill. Schizophr Bull.

[19] Henderson C, Potts L, Robinson EJ (2019). Mental illness stigma after a decade of time to change England: inequalities as targets for further improvement. Eur J Pub Health.

[20] Ilic N, Henderson C, Evans-Lacko S, Thornicroft G. Attitudes towards mental illness. Health Survey Engl. 2014:1–15.

[21] Evans-Lacko S, Rose D, Little K, Flach C, Rhydderch D, Henderson C, Thornicroft G (2011). Development and psychometric properties of the reported and intended behaviour scale (RIBS): a stigma-related behaviour measure. Epidemiol Psychiatr Sci.

[22] Star SA. What the public thinks about mental health and mental illness. Indianapolis, Indiana1952.

[23] Robinson EJ, Henderson C (2019). Public knowledge, attitudes, social distance and reporting contact with people with mental illness 2009–2017. Psychol Med.

[24] Evans-Lacko S, Henderson C, Thornicroft G (2013). Public knowledge, attitudes and behaviour regarding people with mental illness in England 2009-2012. Br J Psychiatry.

[25] Cechnicki A, Angermeyer MC, Bielańska A (2011). Anticipated and experienced stigma among people with schizophrenia: its nature and correlates. Soc Psychiatry Psychiatr Epidemiol.

[26] Mehta N, Clement S, Marcus E, Stona A-C, Bezborodovs N, Evans-Lacko S, Palacios J, Docherty M, Barley E, Rose D, Koschorke M, Shidhaye R, Henderson C, Thornicroft G (2015). Evidence for effective interventions to reduce mental health-related stigma and discrimination in the medium and long term: systematic review. Br J Psychiatry.

[27] Edge D, Grey P (2018). An assets-based approach to co-producing a culturally adapted family intervention (CaFI) with African Caribbeans diagnosed with schizophrenia and their families. Ethn Dis.

[28] Codjoe L, Barber S, Ahuja S, Thornicroft G, Henderson C, Lempp H, N’Danga-Koroma J. Evidence for interventions to promote mental health and reduce stigma in Black faith communities: systematic review. Soc Psychiatr Psychiatr Epidemiol. 2021;18:1–17. 10.1007/s00127-021-02068-y PMC8053235 33866378

[29] Henderson C, Stuart H, Hansson L (2016). Lessons from the results of three national antistigma programmes. Acta Psychiatr Scand.

[30] The Global Anti-Stigma Alliance (GASA) [Available from: https://www.time-to-change.org.uk/about-us/what-we-do/our-global-work/global-anti-stigma-alliance.

