# Supplementary material for: Evaluation of anti-stigma social marketing campaigns in Ghana and Kenya: Time to Change Global
Source: BMC Public Health. 2021 May 8;21:886. doi: 10.1186/s12889-021-10966-8 (PMC8106856; doi:10.1186/s12889-021-10966-8)
Supplement: Supplementary file 1 — Additional file 1: Supplementary 1. Understanding Mental Health Stigmatization and Discrimination Questionnaire September, 2019 [file 12889_2021_10966_MOESM1_ESM.pdf]

PROJECT THINK

INTERVIEWER ID NO.			

SUPERVISOR ID NO			

RESPONDENT ID NO.				

**QUESTIONNAIRE CODE**	
**NUMBER OF HOUSEHOLD IN DWELLING STRUCTURE**	
**NUMBER OF HOUSEHOLD IN DWELLING STRUCTURE SELECTED**	
**IF SUBSTITUTE FOR DWELLING STRUCTURE/HOUSEHOLD**	**WRITE IN**

INTRODUCTION

Good morning/afternoon/evening. My name is …………………… I work for an independent market research company called Consumer Insights Consult Limited. We are currently conducting a study to find out consumer behaviour of people like you. We would be grateful if you assist us in our research. May I assure you that this is a genuine piece of market research. I am not trying to sell you anything and the information that you will provide will be used solely for research purposes.

INTERVIEWER TO EXPLAIN TO RESPONDENT THAT HIS / HER IDENTITY WILL REMAIN CONFIDENTIAL AND THAT CONSUMER INSIGHTS CONSULT COMPLIES WITH THE MARKETING RESEARCH CODE OF CONDUCT

Interviewer's Name………………………………

Supervisor's Name……………………………

Respondent's Name……………………………

Back Checked by ……………………………

Address…………………………………………

Date of Interview ……………………………

Sampling Selection Instructions

Interviewer: Recruit Respondent by Kish Grid. Please tell me the names and ages of all males or females (as required) who are **18- 35** years of age starting from the oldest within this age group.

INTERVIEWER: RECORD THE NAMES OF ALL ADULTS WITHIN THE TARGET AGE GROUPS. RECORD MALE OR FEMALE MEMBERS (AS REQUIRED) FROM THE OLDEST TO THE YOUNGEST WITHIN THE RELEVANT AGE GROUP AND CHOOSE ONE USING THE KISH-GRID (TABLE) BELOW. REMEMBER TO LIST ONLY ONE GENDER IN EACH HOUSEHOLD.

	Name	Age	A	B	C	D	E	F	G	H	J	K
1			1	1	1	1	1	1	1	1	1	1
2			2	1	2	1	2	1	1	2	2	1
3			3	1	2	3	1	1	3	1	2	1
4			2	2	3	3	1	2	4	3	4	4
5			4	2	4	5	5	3	1	5	3	3
6			5	5	5	2	3	4	1	3	2	6

RANDOM SELECTION OF RESPONDENT by Rotating Gender selection

SCREENING QUESTIONNAIRE

S1. **GENDER**: Record the gender of the respondent from observation. **DO NOT ASK**

Male	1
Female	2

S2. Have you participated in any market/social research interview in the past 6 months? **READ OUT**

	**(X)**	
Yes	1	**TERMINATE**
No	2	**CONTINUE TO S3**

S3. Could you please tell me which of the following age groups do you belong?

Below 18 below years	1	**TERMINATE**	**RECORD EXACT AGE**
18 - 25	2	**CONTINUE TO Q1**	
26 - 30	3		
31 - 35	4		
Above 35 years	9	**TERMINATE**	

MAIN QUESTIONNAIRE

Q1. How many family members currently live in this household?

Q2. How many children (less than 16 years old) live in the household? (include all children who stay, eat, and sleep in the household)

Q3. How many adults (16 years old or more) live in the household? (include yourself and all adults who stay, eat, and sleep in the household etc.)

Q4. Does anyone close to you have or have had some kind of mental health problem? Single response

No-one known	1
Immediate family (spouse\sister\brother\parent\child	2
Partner	3
Other family (uncle\aunt\cousin\grandparent etc.)	4
Friend	5
Work colleague	6
Self	7
Other (please specify)	8

The following questions ask about your views in relation to people who have mental health. For the following questions, please respond by choosing one answer.

Q5. Please can you tell me how strongly you agree or disagree with the following statements?

		Agree strongly	Agree slightly	Neither agree nor disagree	Disagree slightly	Disagree strongly	DK
a	In the future, I would be willing to live with someone with a mental health problem	5	4	3	2	1	99
b	In the future, I would be willing to work with someone with a mental health problem	5	4	3	2	1	99
c	In the future, I would be willing to live nearby to someone with a mental health problem	5	4	3	2	1	99
d	In the future, I would be willing to continue a relationship with a friend who developed a mental health problem	5	4	3	2	1	99

Q6. And to what extent do you agree or disagree with the following statements?

		Agree strongly	Agree slightly	Neither agree nor disagree	Disagree slightly	Disagree strongly	DK
a	One of the main causes of mental illness is a lack of self-discipline and will-power	5	4	3	2	1	99
b	There is something about people with mental illness that makes it easy to tell them from normal people	5	4	3	2	1	99
c	We need to adopt a far more tolerant attitude toward people with mental illness in our society	5	4	3	2	1	99
d	People with mental illness don’t deserve our sympathy	5	4	3	2	1	99
e	I would not want to live next door to someone who has been mentally ill	5	4	3	2	1	99
f	It is frightening to think of people with mental problems living in residential neighbourhoods	5	4	3	2	1	99

Q7. And to what extent do you agree or disagree with the following statements?

		Agree strongly	Agree slightly	Neither agree nor disagree	Disagree slightly	Disagree strongly	DK
g	Mental illness is an illness like any other	5	4	3	2	1	99
h	Virtually anyone can become mentally ill	5	4	3	2	1	99
i	The best therapy for many people with mental illness is to be part of a normal community	5	4	3	2	1	99
j	People with mental health problems are far less of a danger than most people suppose	5	4	3	2	1	99
k	People with mental health problems should not be given any responsibility	5	4	3	2	1	99
l	I have very little knowledge about mental illness	5	4	3	2	1	99
m	Most women who were once patients in a mental hospital can be trusted as babysitters	5	4	3	2	1	99

Q8. Can you tell me how strongly you agree or disagree with the following statements? Mental health problems here refer, for example, to conditions for which an individual would be seen by healthcare staff?

		Agree strongly	Agree slightly	Neither agree nor disagree	Disagree slightly	Disagree strongly	DK
a	Most people with mental health problems want to have paid employment	5	4	3	2	1	99
b	If a friend had a mental health problem, I know what advice to give them to get professional help	5	4	3	2	1	99
c	Medication can be an effective treatment for people with mental health problems	5	4	3	2	1	99
d	Psychotherapy (eg. talking therapy or counselling) can be an effective treatment for people with mental health problems	5	4	3	2	1	99
e	People with severe mental health problems can fully recover	5	4	3	2	1	99
f	Most people with mental health problems go to a healthcare professional to get help	5	4	3	2	1	99
g	Mental health problems are genetic – they are passed on through generations	5	4	3	2	1	99
h	Mental health problems can be caused by a curse	5	4	3	2	1	99

Q9. Can you tell me to what extent you agree or disagree that each of the following is a type of mental health problem?

		Agree strongly	Agree slightly	Neither agree nor disagree	Disagree slightly	Disagree strongly	DK
a	Depression	5	4	3	2	1	99
b	Stress	5	4	3	2	1	99
c	Schizophrenia	5	4	3	2	1	99
d	Bipolar disorder (manic-depression)	5	4	3	2	1	99
e	Drug addiction	5	4	3	2	1	99
f	Grief	5	4	3	2	1	99

	**Have you ....**	Yes	No	Don't know
Q10	Met anyone that has talked about their own experience of mental health problems recently?	1	2	3
Q11	Seen any activity or publicity for a mental health campaign in the local community at all recently?	1	2	3
Q12	Read, any news stories or articles about a mental health campaign in newspapers, magazines or online recently?	1	2	3
Q13	Heard any advertising or features on the radio recently for a mental health campaign?	1	2	3
Q14	Seen any advertising for a mental health campaign in any newspapers or magazines press recently?	1	2	3
Q15	Seen any activity or advertising online for a mental health campaign?	1	2	3
Q16	Seen any advertising on TV recently for a mental health campaign?	1	2	3
Q17	Seen any advertising on social media for a mental health campaign?	1	2	3

Interviewer: Please show relevant advert to respondent ad ask the following questions;

		Yes	No	Don't know
Q18	This is a clip of some advertising that may have been played on the radio recently. Do you remember hearing it before?	1	2	3
Q19	This is a picture of some advertising that may have been posted in the local community recently. Do you remember seeing it before?	1	2	3
Q20	This is a picture of some advertising that may have been appeared in the newspapers recently. Do you remember seeing it before?	1	2	3
Q21	This is an advert that may have been appeared online recently. Do you remember seeing it before?	1	2	3

DEMOGRAPHICS

F1. What is your occupation?

Farmer	1
Trader	2
Mason	3
Carpenter	4
Tailor/Seamstress/Fashion designer	5
Hairdresser	6
Teacher	7
Government Employee	8
Worker at a private firm	9
Security	10
Housewife	11
Self-employed/own business	12
Apprentice	13
Student	14
Retired	15
Not working	16
Other, please specify	17

F2. Please, may I know your level of education?

None	1
Kindergarten	2
Primary	3
JSS/JHS	4
Middle	5
SSS/SHS	6
Secondary	7

Vocational/Technical/Comm. Teacher Training/Agricultural	8
Nursing Certificate	9
Post-Secondary Diploma (HND, Teacher training, Nursing, University Diploma)	10
Bachelor degree	12
Post graduate	13
Don't know	14

F3. What is your religious denomination?

Catholic / Anglican	1
Presbyterian, Baptist, Methodist	2
Seventh-Day Adventist (SDA)	3
Pentecostal/ Charismatic	4
Other Christians	5
Islam	6
Traditional	7
No religion	8
Other religion	9

F4. Which of this best describes your marital status?

Married	1
Living with someone in a stable relationship	2
Single/never married	3
Divorced/separated	4
Widowed	5

F5. Ethnicity?

Akan	1
Ashanti	2
Ewe	3
Fanti	4
Ga	5
Ga Adangbe	6
Nzema	7
Kusasi	8
Nanumba	9
Krobo	10
Frafra	11
Akuapem	12
Dagomba	13
Gonja	14
Mamprusi	15
Kasena	16
Efutu	17
Konkomba	18
Other, please specify	19

SOCIAL CLASS MEASUREMENT

INTERVIEWER READ: NOW I WOULD LIKE TO ASK YOU ABOUT YOUR LIVING STANDARD IN THIS HOUSEHOLD. THERE ARE SOME ITEMS WHICH I WOULD MENTION AND YOU EITHER TELL ME ''YES''- IF YOU POSSESS THEM OR ''NO''- IF YOU DON'T POSSESS THEM.

Living Standard Measurement Table

Socio-economic grading via income of household or income of the respondent is not practicable in this part of the world hence the use of various relevant household durables with appropriate weight to each variable in terms of scores as demonstrated below:

Type	Description	Score	Instruction
Ownership	Household help	2	Multiple Answer possible
	Fridge/deep freezer	3	
	Video	1	
	Car	2	
	Colour TV	1	
	Music system	1	
	Air conditioning unit (split)	4	
	Air conditioning	3	
	Satellite dish	3	
	Washing machine	4	
	Black & White TV	1	
	DVD (Digital video disk)	4	
	Cable satellite	2	
	Telephone (land)	3	
	Telephone (mobile)	1	
	Personal driver	2	
	Multiple cars	3	
	Computer	3	
	Computer Laptop	4	
	Generator	4	
Cooking	Gas/Electric Cooker	2	Multiple Answer possible
	Kerosene stove	1	
	Charcoal/wood	0	
Toilet Type	Inside/Outside WC	2	Single Answer
	Pit latrine	1	
	None	0	
Main Water Source	Inside	3	Single Answer
	Outside pipe borne tap	3	
	Borehole	2	
	Well	1	
	Stream	0	

	Description	Score	Instruction
Education of household head	Primary Incomplete	1	Single Answer
	Primary complete	1	
	Secondary Incomplete	1	
	Secondary complete	2	
	University/Polytechnic: OND	3	
	University/Polytechnic: HND	3	
	Post-University Incomplete	4	
	Post University Complete	5	
	Illiterate/None	0	
Residential Area	Low density	3	Single Answer
	Medium density	2	
	High density	1	
Type Of House	Self-occupied bungalow	2	
	Villa	5	

	Flat	3	**Single Answer**
	Duplex	4	
	Mini flat	2	
	Room and parlour	1	
	Room	1	
Occupation	Senior Management/Admin.	5	**Single Answer**
	Managing Director	5	
	Head of department/Senior Manager	4	
	Manager	3	
	Professional e.g. Doctor, Lawyer, Engineers, Surveyors etc.	4	
	Skilled workers (mechanics, tailoring, carpenters, bricklayers)	2	
	Unskilled workers	1	
	Clerical workers	2	
	Unemployed	0	
Lifestyle	Membership of social/recreational club	3	**Multiple Answer possible**
	Travel abroad for holidays	4	
	Read regularly as a habit	2	
	Spend leisure time with friends	1	
	Attend social occasions	1	
	Like modern fashion	1	
**TOTAL SCORE**			

USE THIS TABLE TO INDICATE THE SOCIO-ECONOMIC CLASS OF THE RESPONDENT

AB	70 and above	1
**C_1**	56 – 69	2
**C_2**	35 – 55	3
DE	Under 35	4

CLOSE INTERVIEW AND THANK THE RESPONDENT
