# Supplementary material for: Evaluation of anti-stigma social marketing campaigns in Ghana and Kenya: Time to Change Global
Source: BMC Public Health. 2021 May 8;21:886. doi: 10.1186/s12889-021-10966-8 (PMC8106856; doi:10.1186/s12889-021-10966-8)
Supplement: Supplementary file 2 — Additional file 2: Table S2. Percentage (%) of participants who strongly or slightly agree to the MAKS Items and additional knowledge items by Site and Pre vs. Post. Table S3. Percentage (%) of participants who strongly or slightly agree to the CAMI Items by Site and Pre vs. Post. Table S4. Percentage (%) of participants who strongly or slightly agree to the RIBS IB Items by Site and Pre vs. Post. [file 12889_2021_10966_MOESM2_ESM.pdf]

Table S2: Percentage (%) of participants who strongly or slightly agree to the MAKS Items and additional knowledge items by Site and Pre vs. Post

MAKS- Part A (Knowledge)	Ghana Pre (n=408)	Ghana Post (n=402)	Kenya Pre (n=419)	Kenya Post (n=411)
1. Most people with mental health problems want to have paid employment (True)	44.4	44.8	43.2	52.1
2. If a friend had a mental health problem, I know what advice to give them to get professional help (True)	85.3	85.3	66.1	74.9
3. Medication can be an effective treatment for people with mental health problems (True)	81.4	88.6	80.2	81.8
4. Psychotherapy (e.g., talking therapy or counselling) can be an effective treatment for people with mental health problems (True)	89.0	88.1	79.5	86.4
5. People with severe mental health problems can fully recover (True)	68.1	72.1	57.8	62.8
6. Most people with mental health problems go to a healthcare professional to get help (False)	65.4	76.1	52.0	49.6
Mental health problems are genetic – they are passed on through generations (False)	61.8	54.2	38.2	39.7
Mental health problems can be caused by a curse (False)	84.1	74.1	39.4	31.6
**MAKS- Part B (Agreement as to whether each condition is a type of mental illness)**				
Depression (True)	91.2	89.1	89.5	93.7
Stress (False)	73.5	68.7	86.4	86.6
Schizophrenia (True)	89.5	78.1	85.2	86.6
Bipolar disorder (manic depression) (True)	88.7	87.3	86.4	85.6
Drug Addiction (True)	94.4	93.5	82.6	82.2
Grief (False)	80.6	78.4	59.9	69.1

Table S3: Percentage (%) of participants who strongly or slightly agree to the CAMI Items by Site and Pre vs. Post

CAMI (Attitude)	Ghana Pre (n=408)	Ghana Post (n=402)	Kenya Pre (n=419)	Kenya Post (n=411)
1. One of the main causes of mental illness is a lack of self-discipline and willpower	63.0	58.2	35.6	36.7
2. There is something about people with mental illness that makes it easy to tell them from normal people	77.5	82.1	73.8	67.6
3. We need to adopt a far more tolerant attitude toward people with mental illness in our society	86.5	90.6	76.9	76.9
4. People with mental illness don't deserve our sympathy	36.3	20.7	6.9	11.2
5. I would not want to live next door to someone who has been mentally ill	44.1	48.0	24.8	24.3
6. It is frightening to think of people with mental problems living in residential neighbourhoods	47.8	55.2	26.0	29.7
7. Mental illness is an illness like any other	39.2	39.6	83.1	74.5
8. Virtually anyone can become mentally ill	80.4	88.3	87.4	86.6
9. The best therapy for many people with mental illness is to be part of a normal community	71.3	72.6	73.3	70.6
10. People with mental illness are far less of a danger than most people suppose	58.1	57.5	45.6	46.0
11. People with mental illness should not be given any responsibility	46.1	49.0	42.2	32.9
12. Most women who were once patients in a mental hospital can be trusted as babysitters	22.1	33.1	21.2	24.3

Table S4: Percentage (%) of participants who strongly or slightly agree to the RIBS IB Items by Site and Pre vs. Post

**Intended Behaviour (strongly or slightly agree)**	**Ghana Pre (n=408)**	**Ghana Post (n=402)**	**Kenya Pre (n=419)**	**Kenya Post (n=411)**
Live with	33.6	35.6	51.8	50.6
Work with	29.4	39.3	52.7	53.0
Live nearby	47.6	51.5	55.6	53.5
Continue a relationship with a friend	64.2	76.6	47.7	52.1
